# Child eating disorder examination (ChEDE) interview and child eating disorder examination questionnaire (ChEDE-Q): psychometric properties of the Italian versions

**DOI:** 10.1007/s40519-025-01737-0

**Published:** 2025-03-17

**Authors:** Lucilla Bonvini, Silvia Taddei, Saverio Caini, Simona Calugi, Giulia Bugli, Livio Tarchi, Sara Chiari, Ilaria Galli, Ilenia Giunti, Claudia Marino, Simone Tavano, Giovanni Castellini, Valdo Ricca, Stefano Lucarelli, Riccardo Dalle Grave, Tiziana Pisano

**Affiliations:** 1 Child and Adolescent Psychiatric Unit, Neuroscience and Human Genetics Department, Meyer Children’s Hospital IRCCS, Florence, Italy; 2 Cancer Risk Factors and Lifestyle Epidemiology Unit, Institute for Cancer Research, Prevention, and Clinical Network (ISPRO), Via Cosimo Il Vecchio 2, 50139 Florence, Italy; 3 Department of Eating and Weight Disorders, Villa Garda Hospital, Garda, Italy; 4 Eating Disorders Unit, AUSL Toscana Centro, Florence, Italy; 5 Psychiatry Unit, Department of Health Sciences, University of Florence, Largo Brambilla, 3, 50134 Florence, Italy; 6 Child and Adolescent Psychiatry, AUSL Toscana Centro, Florence, Italy

**Keywords:** Eating disorder behaviours, Children and adolescent psychiatry, Psychometric validation, Psychometric characteristics, Reliability, Validity

## Abstract

**Purpose:**

To examine the psychometric characteristics of the Italian language versions of the child eating disorder examination (ChEDE) interview and child eating disorder examination questionnaire (ChEDE-Q).

**Methods:**

ChEDE (from EDE 17th edition) and ChEDE-Q were first translated, and then administered to 147 patients with eating disorders under the age of 18, along with 80 age-matched controls. Their internal consistency (Cronbach alpha), inter-rater reliability (Spearman rho), short-term (7–23 days) test–retest reliability (Spearman rho), and criterion validity (group differences by Mann–Whitney U) were evaluated.

**Results:**

Patients with eating disorders displayed significantly higher ChEDE/ChEDE-Q scores than age-matched controls, demonstrating the adequate criterion validity of the instrument (all subscales and global scores significant at p < 0.001). Internal consistency was high for all original ChEDE/ChEDE-Q subscales (minimum Cronbach alpha 0.752), apart from Eating Concerns (minimum Cronbach alpha 0.591). Inter-rater reliability was excellent for global ChEDE/ChEDE-Q scores and each subscale (minimum Spearman rho 0.999). Test–retest reliability was excellent for global ChEDE/ChEDE-Q scores and each subscale (minimum Spearman rho 0.791).

**Conclusions:**

The Italian versions of the ChEDE interview and ChEDE-Q exhibited excellent psychometric properties and may, therefore, be recommended for the assessment of Italian patients with eating disorders less than 18 years old, both in clinical practice and research settings.

*Level of evidence III* evidence obtained from cohort or case–control analytic studies.

**Supplementary Information:**

The online version contains supplementary material available at 10.1007/s40519-025-01737-0.

## Introduction

Eating disorders (EDs) are complex and debilitating conditions, often complicated by a longstanding course [1]. Within EDs, Anorexia Nervosa (AN), Bulimia Nervosa (BN), and Binge Eating Disorder (BED) have been described as sharing a common psychopathological core of psychopathology and risk factors [2]. More recently, these three disorders have also been suggested to share common biological factors [3].

EDs are associated with a high mortality rate and typically manifest during late childhood and early adolescence [1, 4, 5]. Children and adolescents are the population most at risk due to the vulnerability of this developmental stage, which fosters psychological and physical maturation, as well as the development of body image, and self-perception [6, 7]. It is estimated that 6–8% of adolescents develop an ED [6]. Recognizing EDs can be challenging for primary care providers, and this challenge may delay the early diagnosis and treatment of EDs.

To address this challenge, specific assessment tools have been developed for adults. In particular, the Eating Disorder Examination (EDE) interview [8] is considered the gold standard for assessing ED psychopathology in adults. EDE includes four subscales (Restraint, Eating Concern, Shape Concern, and Weight Concern), which can be evaluated individually or by a global average [8]. The EDE interview is designed to measure the characteristics and severity of ED psychopathology (binge eating episodes, self-induced vomiting, misuse of laxatives or diuretics, and excessive exercise) and to provide operational diagnoses of EDs [8]. The EDE interview has been validated in several languages, including Italian [9]. In parallel with clinician-administered structured interviews, self-reported questionnaires have also been developed to facilitate ED screening and diagnosis. For instance, the Eating Disorder Examination Questionnaire (EDE-Q) [10] is a self-administered version of the EDE, with scoring similar to the EDE, and with characteristics which have also been compared with those of the EDE [10]. Moreover, similarly to EDE, EDE-Q has been validated in different languages and cultural contexts [11, 12].

However, as previously mentioned, most EDs exhibit symptomatic onset during late childhood and early adolescence, when prompt diagnosis and treatment would be particularly beneficial. For these reasons, the child version of EDE (ChEDE) was first developed [13]. Similarly to EDE and EDE-Q, ChEDE has been translated into other languages and adapted for different cultural contexts, demonstrating adequate psychometric properties [14, 15]. Moreover, ChEDE is available as both a clinician-administered examination (ChEDE), and a self-reported questionnaire (ChEDE questionnaire, ChEDE-Q) [13].

Given the increasing incidence of EDs among adolescents and young adults globally and across Europe [16], the lack of validated instruments to assess EDs in children and adolescents in the Italian context underscores the need for a proper psychometric adaptation and validation of the ChEDE and ChEDE-Q in Italian. Therefore, the primary aim of the present study was to psychometrically validate and assess a translated version of ChEDE and ChEDE-Q.

## Material and methods

### Sample and participants

A total of 227 participants were enrolled. Participants were divided into two groups: patients with ED and healthy controls (HC). All participants were enrolled through a multicenter initiative of different psychiatry services (patients diagnosed with EDs at the following institutions: Meyer Children Hospital IRCCS of Florence, Tuscany Regional Services, Villa Garda Hospital). Demographic information, medical examination, Kiddie-SADS- Present and Lifetime Version (K-SADS-PL) results were collected for all participants.

Inclusion criteria for patients with ED were established as follows: age between 8 and 18 years with diagnoses of AN, BN, or BED according to DSM-5-TR [1]. Exclusion criteria for patients with EDwere previous or current diagnosis of schizophrenia and/or other psychotic disorders, substance abuse or misuse, present pharmaceutical interventions, or medical complications that could affect the validity of given responses or text comprehension.

Inclusion criteria for the group of HC individuals included age between 8 and 18 years, BMI > 17.5, no current or past diagnosis of any ED, as assessed both by K-SADS-PL [17] and clinical unstructured interview.

All participants, those in the group of patients with ED and those in the group of HC individuals, were then administered the Italian versions of the ChEDE and the ChEDE-Q by expert personnel in the field of EDs.

### Ethics approval and informed consent

The study design was reviewed and approved by the Pediatric Ethics Committee of the Tuscany Region (reference N°220/20222 EDE-BA, approved on September 7th, 2022), all participants gave written informant assent, and all parents’ participants gave written informed consent for the use of their anonymous personal data.

### Translation procedure

The original items of ChEDE and ChEDE-Q were first translated by expert clinicians in the field of ED, native Italian speakers with sufficient knowledge of the English language. The translation process was conducted as follows: (1) translation into Italian by a single bilingual individual (SCh); (2) blind back-translation into English by another bilingual individual (TP); (3) discussion of the items by the group of investigators to identify any discrepancies and correct any inconsistencies (ST, RDG, LT, GC, IG, ST); and (4) final approval by a multidisciplinary team of diverse clinical background (dietitians, clinical psychologists, psychiatrists).

The translation process of EDE-Q was not dissimilar to that of EDE. For ChEDE-Q, the courtesy form was replaced by a more age-appropriate personal register (i.e., replacing the Italian ‘lei’ with ‘tu’).

A subgroup of patients was randomly chosen to be administered the ChEDE again to assess test–retest reliability (time to retest: one to three weeks). This follow-up interview was administered before any treatment was offered by other interviewers, who were blind to the initial ChEDE scores. Finally, a second subgroup was randomly chosen to be recorded, and these clinical records were analyzed again by different clinicians to assess inter-rater reliability.

### Statistical analysis

Confirmatory factor analysis was adopted to confirm the original four-factor structure of the ChEDE and ChEDE-Q (subscales Restraint, Eating Concern, Weight Concern, and Shape Concern) [18]. This confirmatory analysis was conducted in R 4.3.3 [19] with the support of *lavaan* [20]. A Diagonally Weighted Least Squared estimator (DWLS) was adopted as Maximum Likelihood (ML) estimators are less accurate for ordinal data [21]. The difference between perfect fit and the observed model was estimated with *χ*^*2*^.

Three indices of fit were derived: Comparative Fit Index (CFI), Tucker-Lewis Index (TLI), and root mean square error of approximation (RMSEA). In general, a satisfactory fit would require the p value associated with *χ*^*2*^ to be above 0.05 (not to reject the null hypothesis of perfect fit), CFI above 0.96, TLI above 0.90, and RMSEA below 0.08 (RMSEA p value tests the hypothesis that RMSEA is less or equal to 0.05, and should thus also not be significant) [22].

Internal consistency was calculated by Cronbach’s [23], both for the four subscales and the global score. Criterion validity was measured by assessing group differences using the Mann–Whitney U test, with the aim to reach a satisfactory differentiation between patients with ED and HC (higher scores in patients with ED) [24, 25]. Additionally, factor covariance between the four subscales was computed [24], along with the Spearman correlation coefficient between each subdomain of ChEDE and ChEDE-Q, as well as their global scores.

To measure inter-rater reliability, a random sample of fourteen patients was assessed by two interviewers independently, based on audio recordings of original clinical interviews. To explore test–retest reliability, Spearman's rank correlations were computed within a random subset of twenty inpatients interviewed at baseline and again at follow-up interviews (one to three weeks after the first evaluation).

As patients with AN were the most represented diagnostic group within EDs, control analyses were performed restricting the sample to patients with AN only (criterion validity, confirmatory factor structure, convergent validity).

## Results

### Descriptive statistics

A total of 227 participants were enrolled. The youngest participant was 8 years old, and the oldest 18 years old. Of these, 147 subjects (64.76%) were patients with ED, and 80 (35.24%) individuals were HC. On average, HCs were slightly younger (Hedges’ g 0.703), had higher BMI (Hedges’ g -0.149) and were more likely to be males. A trend for BMI differences between patients with ED and HC was observed even after correcting for sex (males with ED reported higher BMI than male HC: 22.77 ± 13.77 vs. 19.48 ± 2.34; females with ED reported lower BMI than female HC: 18.45 ± 5.32 vs. 19.39 ± 2.74; sex interaction term with BMI by analysis of variance—ANCOVA, F: 3.751, p 0.054). Eighteen out of 147 (12.24%) with ED were under pharmacological treatment at the time of enrolment: eight (relative frequency, 44.44%) were undergoing treatment with an SSRI, four (22.22%) with an antipsychotic agent, another four (22.22%) with benzodiazepines, one (5.55%) with mood stabilizers, and one (5.55%) with both SSRI and an antipsychotic agent. See Table 1 for further details.

**Table 1:** Sample descriptives

	Patients with ED (n = 147)	HC (n = 80)	Effect size	Mann–Whitney p-value	χ^2^ p-value
Age	14.75 ± 1.97	13.21 ± 2.53	0.703	< 0.001	*/*
BMI (kg/m^2)^	18.66 ± 5.96	19.42 ± 2.60	-0.149	< 0.001	*/*
Sex	♂ = 7, 4.76% ♀ = 140 95.24%	♂ = 25, 31.25% ♀ = 55 68.75%	0.364	*/*	< 0.001
Gender	M = 7, 4.76% F = 140, 95.24%	M = 25, 31.25% F = 55, 68.75%	0.364	*/*	< 0.001
DSM-5-TR Diagnosis	AN = 105, 71.43% BN = 27, 18.37% BED = 15, 10.20%	*/*		*/*	*/*

This table compares demographic and clinical characteristics between patients with ED and HC, including age, BMI, and gender distribution. It also provides the effect sizes and statistical significance of these differences

Mean values ± standard deviation*.* Effect size of mean difference for continuous variables in Hedges’ g*.* Effect size of mean divergence for categorical variables in Cramer’s V

*AN *Anorexia Nervosa, *BED* Binge Eating Disorder, *BN* Bulimia Nervosa, *ED* eating disorders, *HC* Healthy Controls, *DSM* Diagnostic and Statistical Manual, *F* Feminine, *M* Masculine

### Internal consistency, criterion validity

The translated questionnaire exhibited significant evidence in favor of the original four factor model (ChEDE: χ^2^ 110.806 p 0.999, CFI 1.000, TLI 1.039, RMSEA 0.000 p 0.999; ChEDE-Q χ^2^ 166.775, p 0.970, CFI 1.000, TLI 1.014, RMSEA 0.000 p 0.999). Patients with ED reported higher scores across all domains and across both global scores (maximum Mann–Whitney p-value < 0.001). See Table 2 for further details.

**Table 2:** Internal consistency, criterion validity

	Patients with ED (n = 147)	HC (n = 80)	Cronbach α ^a^	Mann–Whitney p-value
ChEDE Restraint	3.51 ± 1.28	0.01 ± 0.05	0.818	< 0.001
ChEDE Eating Concern	4.03 ± 1.72	0.00 ± 0.00	0.591	< 0.001
ChEDE Weight Concern	4.73 ± 1.37	0.03 ± 0.15	0.760	< 0.001
ChEDE Shape Concern	5.02 ± 1.26	0.04 ± 0.15	0.882	< 0.001
ChEDE Global Score	4.32 ± 1.20	0.02 ± 0.08	0.918	< 0.001
ChEDE-Q Restraint	4.01 ± 1.58	0.01 ± 0.04	0.832	< 0.001
ChEDE-Q Eating Concern	3.28 ± 1.29	0.03 ± 0.17	0.611	< 0.001
ChEDE-Q Weight Concern	4.44 ± 1.38	0.07 ± 0.19	0.752	< 0.001
ChEDE-Q Shape Concern	4.93 ± 1.26	0.11 ± 0.39	0.878	< 0.001
ChEDE-Q Global Score	4.16 ± 1.18	0.05 ± 0.17	0.917	< 0.001

This table presents the internal consistency of different subscales of the ChEDE and its self-report version for patients with ED and healthy controls (ChEDE-Q), showing high Cronbach's alpha values and significant differences

Mean ± Standard Deviation

*ChEDE* Child Version, Eating Disorder Examination; *ChEDE-Q* Child Version, Eating Disorder Examination Questionnaire, *HC* Healthy Controls

a = computed within patients with ED only

### Factor covariance, convergent validity and test reliability

All four subscales were found to have high covariance (minimum estimate 0.917 for ChEDE, 0.911 for ChEDE-Q, p < 0.001; Table 3 and Table 4). A high correlation between ChEDE and ChEDE-Q domains was observed (minimum Spearman rho 0.318, p < 0.001; Table 5).

**Table 3:** Factor covariance, ChEDE

	ChEDE Restraint	ChEDE Eating Concern	ChEDE Shape Concern	ChEDE Weight Concern
ChEDE Restraint	/	0.870 (p < 0.001)	0.706 (p < 0.001)	0.731 (p < 0.001)
ChEDE Eating Concern	0.870 (p < 0.001)	*/*	0.887 (p < 0.001)	0.866 (p < 0.001)
ChEDE Shape Concern	0.706 (p < 0.001)	0.887 (p < 0.001)	*/*	
ChEDE Weight Concern	0.731 (p < 0.001)	0.866 (p < 0.001)	0.876 (p < 0.001)	*/*

This table shows the correlations between different subscales of the ChEDE, indicating strong interrelationships among these factors

*ChEDE* Child Version, Eating Disorder Examination

**Table 4:** Factor covariance, ChEDE-Q

	ChEDE-Q Restraint	ChEDE-Q Eating Concern	ChEDE-Q Shape Concern	ChEDE-Q Weight Concern
ChEDE-Q Restraint	/	0.652 (p < 0.001)	0.701 (p < 0.001)	0.744 (p < 0.001)
ChEDE-Q Eating Concern	0.652 (p < 0.001)	*/*	0.849 (p < 0.001)	0.928 (p < 0.001)
ChEDE-Q Shape Concern	0.701 (p < 0.001)	0.849 (p < 0.001)	*/*	
ChEDE-Q Weight Concern	0.744 (p < 0.001)	0.928 (p < 0.001)	0.972 (p < 0.001)	*/*

This table presents the correlations between subscales of the ChEDE-Q, showing similarly high correlations, suggesting strong consistency between these dimensions in the self-report format

*ChEDE-Q* Child Version, Eating Disorder Examination Questionnaire

**Table 5:** – Convergent Validity, clinician-administered vs self-report

	ChEDE-Q Restraint	ChEDE-Q Eating Concern	ChEDE-Q Shape Concern	ChEDE-Q Weight Concern	ChEDE-Q Global Score
ChEDE Restraint	0.435 (p < 0.001)	/	*/*	/	/
ChEDE Eating Concern	/	0.318 (p < 0.001)	/	*/*	/
ChEDE Shape Concern	/	*/*	0.612 (p < 0.001)	*/*	*/*
ChEDE Weight Concern	/	*/*	*/*	0.520 (p < 0.001)	*/*
ChEDE Global Score	/	/	/	*/*	0.720 (p < 0.001)

This table compares the correlations between ChEDE and ChEDE-Q subscales, demonstrating convergent validity through significant positive correlations

Spearman’s rho coefficients. Computed within patients with ED only (n = 147)

*ChEDE* Child Version, Eating Disorder Examination, *ChEDE-Q* Child Version, Eating Disorder Examination Questionnaire

Test–retest reliability and inter-rater reliability were found to be excellent (inter-rater reliability: minimum Spearman rho 0.999; test–retest reliability: minimum Spearman rho 0.791; Table 6).

**Table 6:** Test reliability

	Inter-rater sample (n = 14)^a,b^	Inter-rater reliability Spearman rho	Test–retest sample (n = 20)^a,c^	Test–retest reliability Spearman rho
ChEDE Restraint	4.06 ± 2.12	0.999	4.64 ± 1.66	0.791
ChEDE Eating Concern	3.14 ± 1.50	0.999	3.57 ± 1.44	0.974
ChEDE Shape Concern	4.94 ± 1.34	0.999	5.25 ± 1.22	0.959
ChEDE Weight Concern	4.77 ± 1.50	0.999	5.05 ± 1.32	0.942
ChEDE Global Score	4.23 ± 1.35	0.999	4.63 ± 1.19	0.853

This table reports on inter-rater and test–retest reliability for the ChEDE, showing very high Spearman's rho coefficients, indicating strong reliability in different contexts

Mean ± Standard Deviation

*ChEDE* Child Version, Eating Disorder Examination

a = computed within patients with ED only

b = Mean ± Standard Deviation as measured by original rater

c = Mean ± Standard Deviation as measured by baseline examination

### Control analysis, patients with AN

The original four factor model was confirmed also when restricting analyses to patients with AN alone (ChEDE: χ^2^ 98.520 p 0.999, CFI 1.000, TLI 1.070, RMSEA 0.000 p 0.999; ChEDE-Q χ^2^ 151.469, p 0.997, CFI 1.000, TLI 1.037, RMSEA 0.000 p 0.999). Higher reliability was observed in patients with AN in comparison to patients with ED overall (minimum Cronbach α = 0.855; see Supplementary Materials Table S1). Convergent validity was confirmed also when restricting analyses to patients with AN only (patients with AN reported higher scores than HC across all domains, as well as ChEDE/ChEDE-Q global scores; see Supplementary Materials Table S1).

## Discussion

The present study examined criterion validity, internal consistency, inter-rater and test–retest reliabilities of the Italian Version of ChEDE and ChEDE-Q. The findings confirmed the strong psychometric properties of the instruments and reinforced their potential utility for clinical application.

Cronbach’s alpha coefficients for the four original subscales were high, consistent with previous studies in other target languages and conducted in clinical samples [13], demonstrating strong potential for internal consistency of the selected psychometric instrument. Furthermore, its inter-rater reliability, measured using both Spearman’s correlation coefficients, was excellent across both global and subscale ChEDE scores, and test–retest reliability was also satisfactory.

The ability of ChEDE to discriminate between patients and age-matched controls was confirmed, supporting its role alongside ChEDE-Q in clinical practice. Interestingly, global average scores between ChEDE and ChEDE-Q did not show appreciable differences, suggesting that self-administration may be a viable option for patients under 18 years old. Although future studies might attempt to evaluate the superiority or non-inferiority of one instrument over the other (ChEDE vs ChEDE-Q) in specific populations (for instance, early vs late adolescence), the current study suggests that self-administered psychometric questionnaire may appropriately assess ED-specific psychopathological features in minors. Self-administered questionnaires may be less cumbersome and more widely administered in comparison to structured clinical interviews, while also potentially more cost-effective. For these reasons, the authors suggest ChEDE-Q to be adopted as a first choice for the wider assessment of ED psychopathology in minors.

The results of this study have significant clinical implications. First, they highlight the importance of using rigorous tools like ChEDE for the valid assessment of ED psychopathology for children and adolescents, as with adults. Additionally, the significant differences between patients and control scores further emphasize the strong criterion validity of current instruments, with significant implications for early ED identification and treatment in children and adolescents. Recognizing and addressing these measured altered eating attitudes and behaviors carries the potential to prevent the progression of the specific diagnosis [26], reduce the likelihood of diagnostic crossover [27], and, therefore, improve overall treatment outcomes.

### Strength and limits

The strengths of this study lay in the strong psychometric properties of the proposed ChEDE and ChEDE-Q, the inclusion of both inpatients and outpatients, the inclusion of age-matched controls, and the fact that data was gathered from three different ED Units across Italy.

The main limitation of the study concerns the sample composition. Our sample consisted of patients from all diagnostic categories of EDs. It did not assess the validity and reliability of ChEDE within distinct patient subgroups other than AN (e.g., BN, and BED). Furthermore, the average age of our sample was 14 years, ranging from 8 to 18. A high heterogeneity due to age may be described, and future studies might be interested in characterizing the longitudinal clinical trajectory in criterion validity for ChEDE derived diagnoses in longitudinal designs.

Although the current sample size far exceeds what was previously reported in other validation studies for ChEDE [14], the number of males in the ED group was small, which makes it harder to apply these findings to the broader male population. To make the results more valid and useful, future research should include larger and more diverse groups of participants [28], allowing the findings to be tested and confirmed across different demographic groups [29]. Finally, the sample of patients enrolled to evaluate either inter-rater or test–retest reliability was limited, and the two clinicians scoring recorded sessions belonged to the same service. This limitation could bias results, potentially increasing the estimated inter-rater validity.

### What is already known on this subject?

The ChEDE and ChEDE-Q can play a crucial role in helping clinicians accurately diagnosing and tracking the severity of symptoms across various types of EDs. Their implementation in clinical practice may significantly improve the diagnosis and treatment of early-onset EDs, as well as provide more tailored treatment strategies for Italian-speaking patients.

### What this study adds?

The present study introduces a new validation of ChEDE and ChEDE-Q in the Italian language. These tools may enhance the ability to accurately diagnose and monitor the severity of ED psychopathology across several diagnostic categories within EDs.

## Conclusion

The present study highlights the significant potential for Italian-validated psychometric questionnaires to diagnose children and adolescents exhibiting divergent clinical presentations. The hereby validated questionnaires, ChEDE and ChEDE-Q, offer the potential to differentiate patients by ED psychopathology, being capable to differentiate between patients with ED and HC, while also emphasizing the severity and diverse range of symptoms across EDs. EDE and EDE-Q have previously proven to be reliable and consistent tools for assessing EDs, and their validation for minors carries significant clinical implications for the diagnosis and treatment of EDs in this vulnerable population.

## Supplementary Information

Below is the link to the electronic supplementary material.

Supplementary file 1.

## Data Availability

The dataset generated during the current study is available from the corresponding author upon reasonable request.
