# Supplementary material for: Child eating disorder examination (ChEDE) interview and child eating disorder examination questionnaire (ChEDE-Q): psychometric properties of the Italian versions
Source: Eat Weight Disord. 2025 Mar 17;30(1):26. doi: 10.1007/s40519-025-01737-0 (PMC11914358; doi:10.1007/s40519-025-01737-0)
Supplement: Supplementary file 1 [file 40519_2025_1737_MOESM1_ESM.docx]

**Supplementary Materials**

| **Table S1 – Control analysis, convergent validity, patients with AN vs HC** | | | | |
| --- | --- | --- | --- | --- |
|  | **Patients with AN**  **(n = 105)** | **HC**  **(n = 80)** | **Cronbach α ^a^** | **Mann-Whitney p-value** |
| ChEDE Restraint | 3.49 ± 1.22 | 0.01 ± 0.05 | 0.941 | <0.001 |
| ChEDE Eating Concern | 4.30 ± 1.55 | 0.00 ± 0.00 | 0.862 | <0.001 |
| ChEDE Weight Concern | 4.73 ± 1.36 | 0.03 ± 0.15 | 0.984 | <0.001 |
| ChEDE Shape Concern | 5.03 ± 1.36 | 0.04 ± 0.15 | 0.954 | <0.001 |
| ChEDE Global Score | 4.39 ± 1.17 | 0.02 ± 0.08 | 0.985 | <0.001 |
| ChEDEQ Restraint | 4.27 ± 1.40 | 0.01 ± 0.04 | 0.942 | <0.001 |
| ChEDE-Q Eating Concern | 3.23 ± 1.20 | 0.03 ± 0.17 | 0.855 | <0.001 |
| ChEDE-Q Weight Concern | 4.48 ± 1.29 | 0.07 ± 0.19 | 0.950 | <0.001 |
| ChEDE-Q Shape Concern | 4.99 ± 1.15 | 0.11 ± 0.39 | 0.983 | <0.001 |
| ChEDE-Q Global Score | 4.24 ± 1.05 | 0.05 ± 0.17 | 0.985 | <0.001 |

This table presents the internal consistency of different subscales of the ChEDE, and its self-report version for patients with AN and healthy controls (ChEDE-Q), showing high Cronbach's alpha values and significant differences.

*Note:* Mean ± Standard Deviation.

*Legend:* ChEDE – Child Version, Eating Disorder Examination; ChEDE-Q – Child Version, Eating Disorder Examination Questionnaire, HC = healthy controls.

a = computed within patients with AN only
